# Association of follicle stimulating hormone receptor promoter with ovarian response in IVF-ET patients

**Published:** 2015-11

**Authors:** Wang Dan, Gao Jing, Xia Liangbin, Zhang Ting, Zeng Ying

**Affiliations:** *Department of Obstetrics and Gynecology，Ren Ming Hospital of Wu Han Univeristy，Wu Han，China.*

**Keywords:** *Follicle stimulating hormone receptor*, *Ovarian granulosa cells*, *Infertility*, *Ovarian response*, *Promoter*

## Abstract

**Background:**

Poor ovarian response phenomenon has been observed in some of the in vitro fertilization-embryo transfer patients. Some investigations found that follicle stimulating hormone receptor *(FSHR)* gene plays a role in the process, but no direct evidence shows the correlation between genotypes of *FSHR* and ovarian response.

**Objective:**

Exploring the molecular mechanism behind the mutation of *FSHR* promoter association with ovarian granulosa cells and poor ovarian response.

**Materials and Methods:**

This cross sectional study was performed using 158 women undergoing the controlled short program ovarian stimulation for IVF treatment. The 263 bp DNA fragments before the follicle stimulating hormone (FSH) receptor 5' initiation site were sequenced in the patients under IVF cycle, 70 of which had poor ovarian response and 88 showed normal ovarian responses.

**Results:**

With a mutation rate of 40%, 63 in 158 cases showed a 29^th^ site G→A point mutation; among the mutated cases, the mutation rate of the poor ovarian responders was significantly higher than the normal group (60% versus 23.9%; χ2=21.450, p<0.001). Besides, the variability was also obvious in antral follicle count, and ovum pick-ups. The estradiol peak values and the number of mature eggs between the two groups had significant difference. However, there was no obvious variability (t=0.457, p=0.324) in the basic FSH values between the two groups (normal group, 7.2±2.3 U/L; mutation group, 7.1±2.0 U/L).

**Conclusion:**

The activity of *FSHR* promoter is significantly affected by the 29^th^ site G→A mutation that will weaken promoter activity and result in poor response to FSH.

## Introduction

Poor response in ovary, characterized by the inferior capacity of acquiring the ovum and low level of estradiol (E2) after ovarian stimulation, accounts for the poor fertility in women. Follicle stimulating hormone (FSH) shows its biological effect on the ovary by combining with the specific follicle stimulating hormone receptor (FSHR) which will then activating the adenyl cyclase/cyclic adenosine monophosphate (cAMP) (1). FSHR exist exclusively on the surface of the membrane of the women’s ovarian granulosa cells and the men’s testicular sertoli cells. As the initial spot for transcription, *FSHR* promoter area can activate RNA polymerase to combine seamlessly with DNA template, to affect follicular development and the hormopoiesis of sex hormones. Previous studies in human *FSHR* promoter has revealed five transcriptional starting sites at positions -184,-114, -99, -83, and -79 and also found that the integrity of the region from -1 to -225 sited in the 5’ flanking region of the human *FSHR* is needed for the maximal promoter activity (2, 3).

However, the mechanisms underlying the relationship between the integrity of the 263 bp DNA fragments sited before the 5’ flanking region of the human *FSHR* and the poor ovarian response in in vitro fertilization-embryo transfer (IVF-ET) cycle are not thoroughly understood. 

In this report, we sequenced the 263 bp DNA fragments sited before the 5’ flanking region of the human *FSHR* in the patients with normal or poor ovarian response, to discuss the mechanism of the poor ovarian response in IVF-ET cycle.

## Materials and methods

This cross sectional study was performed on 158 women undergoing the controlled short program ovarian stimulation for IVF treatment in Reproductive Center of Renmin Hospital of Wuhan University from April 2005 to March 2008. All procedures were carried out with the informed consent of all study subjects and approved by the ethics committee of the Renmin Hospital of Wuhan University, Wu Han, China.

Seventy poor ovarian response women with only male or tubal factor infertility who underwent the controlled short program ovarian stimulation for IVF treatment were prospectively recruited in this study as the case group and eighty women with normal ovarian response underwent the controlled short program ovarian stimulation for IVF treatment as the control group.

All women using steroid hormone in the last three months or suffering from endocrine diseases were excluded.

**Materials**

DNA was extracted using genomic DNA extraction kit (American BioSciences, Inc, United States), and purified using multifunction DNA purification and reclamation kit (Bataike Biotechnology Limited Company in Peking). The primer was made by Boya Company in Shanghai (diluted to10 pmol/µL to use). Sequencing was made by Boshang Biotechnology Company in Peking. Ex taq enzyme, 10×PCR Buffer, dNTPs, and Long and AccuratePCR Kit were supplied by Baosheng Company in Dalian. The instruments used for the study were; 164-5050 electrophoresis apparatus, 165-3301 perpendicular electrophoresis bath, 170-3930 transfer modular, My Cycler PCR Instrument (Bio-Rad Company), gelatum imaging analytical system BTS-20.M (UVItec Company of America), and ACS180SE Auto chemiluminescence immune assay analyzer (Bayer Company of America).

**Methods**

1) Determination of sexual hormone: on the second day of menstruation, fasting blood was collected and FSH was measured. Peak E2 levels on the day of HCG administration were determined as indicators of ovarian response. The venous blood was conserved at -20^°^C. Then the genomic DNA of white blood cell was extracted to detect the genotype of the 263 bp DNA fragment before the 5’ flanking region of the human *FSHR*.

2) Detecting the number of the AFC: the AFC was counted on the second day of menstruation by transvaginal Doppler ultrasound instrument.

3) Genotyping 263 bp DNA fragment before the 5’ flanking region of the human *FSHR*.

4) According to the literature (1, 3), a pair of primer was designed: the sequence of the sense oligonucleotide primer was 5′-TATTCCAGACATGCCTAATGG-3′ and the antisense oligonucleotides primer was 5′-AATTATGCATCCATCCACCTG-3′. The PCR volume was 50 µl containing 4 µl of genomic DNA, 5 µl of 10×PCR buffer, 4 µl of dNTP mixture, 2.5 µl of each primer (10 pmol/μl), 0.25 µl of Ex Taq enzyme, 1.5 µl of MgCl2 (25 mmol/L) and finally double distilled water was added till the whole mixture reached 50 µl. The requirements for denaturation, annealing and elongation were respectively at 94^°^C for 1 min, 60^°^C for 1 min, 72^°^C for 1 min in 30 cycles. The product of PCR was authenticated initially by agarose gel electrophoresis of 10 g/L and reclaimed by the gel reclaim kit. To sequence the gene order of the 263 bp DNA fragment before the 5’ flanking region of the human *FSHR*.

**Statistical analysis**

Data processing and statistical analysis were performed under Statistical Package for the Social Sciences, version 11.5, SPSS Inc, Chicago, Illinois, USA (SPSS software). All of the measurement data was expressed by Mean±**SD** and ratio. The simplex factor analysis of the enumeration data was detected by χ^2^ test. The measurement data was detected by Student’s *t*-test. If p<0.05, the discrepancy was considered to have statistical significance.

## Results

The mean age in the case group was 27±5 years old and the average body mass index was 22.59 ± 3.64 kg/m^2^. The control group has a mean age of 30±4 years old, and the mean body mass index was 20.52±2.22 kg/m^2^. These parameters were not significantly different between two groups (p=0.325 and p=0.417, age and body mass index respectively).

**Comparison of the clinical parameters**

The mean FSH level in the two groups had no statistical significance (t=0.457, p=0.324), while the number of the antral follicle, gaining ovarian follicle, and the peak value of serum E2 on the day of injection of HCG were statistically different (χ^2^ were 42.51, 40.35, and 23.74 respectively, p<0.0001). Compared with the controls, the clinical parameters in the poor ovarian response group were significantly fewer (Table I).

**Comparison of the amplified** ***FSHR*** **promoter region**

**Figure 1:**
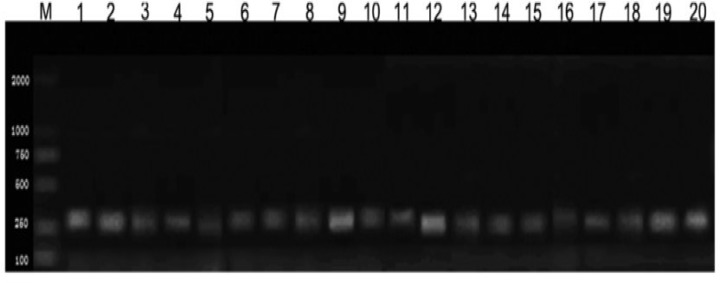
The electrophoresis of the PCR product of the *FSHR* promoter region from the genomic DNA of each group

Initially, 158 cases of genomic DNA were extracted from 158 specimens successfully. Basing on the gene bank data and references (3), promoter fragment P (-263/-1) was amplificated and separated by PCR technique on the template of genomic DNA. The 263 bp DNA fragment was attained by agarose gel electrophoresis identification, reclamation and depuration (Figure 1).

**Figure 2:**
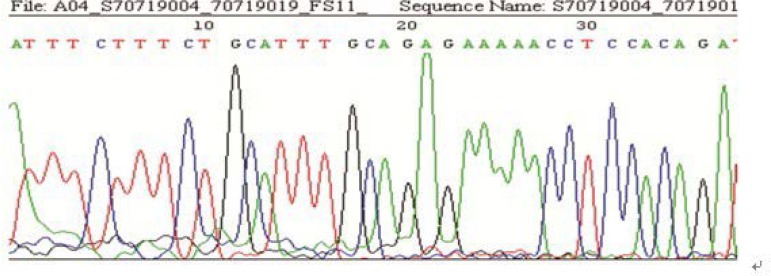
The graph of a homozygous mutation in the *FSHR* sequence (Sample 11)

**Figure 3:**
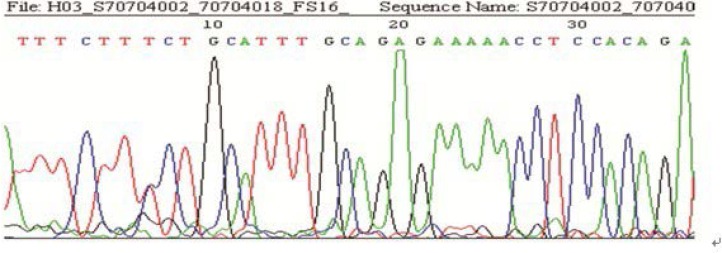
The graph of a heterozygous mutation in the *FSHR* sequence (Sample 16)

The results of sequencing in the 158 cases showed, 63 with the mutation of G→A at the site -29 (among them, 10 were heterozygote; 53 were homozygote), and 95 without mutation. The rate of mutation was 40% (63/158) (Figure 2-3). Comparing the rate of gene mutation of the two groups, the differences were statistically significant (χ^2^=21.450, p<0.001). The A/A genotype had a high incidence rate in both groups and it was even higher in the poor ovarian responders (Table II).

Ovarian response ability in different genotypes: Student’s *t* test was used in the measurement data analysis. The basic level of FSH in G/G genotype was 7.2±2.3 U/L, while in G/A and A/A genotypes were 7.1±2.0 U/L. There were no statistical significant difference between the two groups (p=0.336). However, the number of the antral follicle count, gaining ovarian follicle and mature ovum and the peak value of serum E2 on the day of injection of HCG were statistically higher in G/G genotype than in G/A and A/A genotypes (Table III), (p<0.0001). It indicates that the ovarian response ability of the group with genetic mutation was poorer than the normal group (Table III).

**Table I:** The clinical parameter comparison between the case and control groups

**Groups**	**B** **asic** **FSH** **(U/L)**	**No. of antral** **follicle**	**No. of gaining** **ovarian follicle**	**Serum ** **e** **stradiol** ** (pmol/L)**
Case (n=70)	7.20±2.90	4.70±1.10	4.20±1.30	980±221
Control (n=88)	7.00±2.50	13.50±1.50	12.60±1.30	2560±584
p-value*	0.324	<0.0001	<0.0001	<0.0001

Case group: poor ovarian response; FSH: follicle stimulating hormone, All data are presented by mean ± SD;

* Student’s *t* test

**Table II:** Comparison of the rate of mutation in the *FSHR* promoter region in the two groups

**Groups**	**Genotype mutations in the ** ***FSHR*** ** promoter (n%)**		
	**G/G genotype**	**G/A genotype**	**A/A genotype**
Case	28 (40.0)	6 (8.60)	36 (51.40)
Control	67 (76.10)	4 (4.60)	17 (19.30)

**Table III:** Comparison of the ovarian response in the normal group and the genetic mutation group

**Genotype**	**Cases (n)**	**Basic FSH (U/L)**	**Antral follicle count**	**Gaining ovarian follicle**	**Mature ovum**	**E2 (pmol/L)**
G/G	95	7.20±2.30	14.20±1.30	14.00±1.20	13.60±1.20	2865±557
G/A&A/A	63	7.10±2.00	4.50±0.80	4.50±1.10	4.30±0.90	880±211
p-value		0.336	<0.001	<0.001	<0.001	<0.001

All data were presented by mean ± SD. Student’s *t* test

FSH: follicle stimulating hormone, E2: Estradiol

## Discussion

During superovulation, the ovarian response to drugs is variable among individuals. Clinically the ovarian responses to the stimulation of FSH could be excessive, normal or poor. Among them, poor ovarian response with the incidence rate of 9-26% was a tough problem in superovulation of IVF which could lead to the treatment cycle cancellation, characterized by failure of attaining the ideal efficacy of superovulation, fewer number of gaining ovarian follicle after superovulation and low level of E2. Although the ovarian over response might gain more ovum and embryo, the over response might cause ovarian hyper-stimulation syndrome (OHSS) which is a potential life threatening complication.

The main reason of ovarian poor response was its poor reaction to FSH. It was verified that the stable state receptor (*FSHR*) was activated by the conjunction of FSH and several Leucine-rich repeated sequence of the extracellular region of *FSHR*. Then the activated *FSHR* stimulated the couple guanosine binding protein (Gs protein) situated on the cell endomembrane. The activated Gs protein stimulated the adenylate cyclase which could facilitate the production of cAMP. As an intracytoplasm second messenger, cAMP stimulated protein kinase A to facilitate proteinum phosphorylation and showed its physiological effect (5). The research of Cai *et al*, suggested that the mRNA expression of FSHR on the granular cell of the patients with the ovarian poor response was lower than that of the normal people (6). 

So did the expression of FSHR protein on the granular cell, indicating the effect was decreased in the patients of the poor ovarian response, when the FSH and FSHR were integrated. They also found that the level of FSHR protein has a positive correlation with the peak value of E2 and the number of the mature ovum. In this research we found that the peak value of E2 and the number of the mature ovum in the group of ovarian poor responders were lower than its corresponding values in the normal group. The two outcomes were consistent. In this research the basic FSH between the poor ovarian response group and the normal controls had no difference. While the incidence rate of genetic mutation of G→A at the -29 site of *FSHR* promoter region had significant differences. This indicated that the secretory levels of FSH in vivo of the patients with the poor ovarian response were normal and the poor ovarian response might be caused by the genetic mutation of the *FSHR* promoter.

*FSHR* promoter started the genetic transcription and provoked the post-genetic transcriptional changes of FSHR, which affects growth and maturity of ovarian follicle and generator cells. Meanwhile *FSHR* promoter could regulate the synthesis of steroid hormone. A meta-analysis on the sequence of human promoters showed that the polymorphism of mononucleotide had existed in about 35% of the gene, indicating that the variance reach to 1/3 would change the expression of gene.

Simoni *et al*. had found that various kinds of single nucleotide polymorphism (SNP) was discovered in the core promoter region and coding region of the *FSHR* gene (7). A frequent SNP of *FSHR* core promoter was sited at -29 induced a potential mutation of G→A in the E-26-specific transcription factor, GGAA-binding domain, with its 30% incidence rate in the crowd. Wunsch *et al*, through their research on the genomic DNA of the infertility patients undertaking the IVF treatment for the reasons of the male factor or tubal factor with controlled ovarian hyperstimulation, found two SNP and three mutations in the region of *FSHR* promoter sited at -29, -37, -114, -123 and -138 of the upriver coding region of the transcription initiation (4). In the 158 cases of this research, 63 were founded with the mutation of G→A sited at -29 and the incidence rate was 40%. Among them 10 cases were heterozygote and 53 cases were homozygote. 

The differences of the basic level of the serum FSH between the poor ovarian response group and the normal group were of no statistical significance. These results were in accordance with the references. What’s more, it was observed that the incidence rate of the mutation of G→A at the -29 site of the poor ovarian response group was much higher than the normal group. It indicated that the -29 site might affect the activity of the *FSHR* promoter markedly and the mutation of G→A might weaken the activity of the promoter, then the translation process of *FSHR* was hindered and the proteinaceous production of FSHR was reduced. All of above might finally cause the poor response of granule cells to FSH.

We researched PubMed and SCI database to compare the relationship between *FSHR* promoter genotype mutation and ovarian response in different ethnic groups. A study carried in India revealed almost 72% of subjects with the AA genotype at position -29 of *FSHR* gene were poor ovarian responders (p<0.05), owing to reduced receptor expression, similar to our results (8). However, another study performed in Germany showed an opposite outcome, indicating no correlation between basal FSH serum levels or ovarian response and the SNP at position -29 (9). These three different conclusions indicate the relation between *FSHR* promoter gene and ovarian response differs in different ethnic groups, which shows the need for more evidences.

*FSHR* genotypes were significantly associated with ovarian response to drug stimulation. *FSHR* genotype analysis could be informative for ovarian stimulation outcome and the selection of the proper stimulation protocol, to ensure a sufficient number of mature oocytes for IVF/ICSI. What is more, the A/A and G/A genotypes could be used as a potential marker to predict poor ovarian response.
